# Peripheral PD-1^+^NK cells could predict the 28-day mortality in sepsis patients

**DOI:** 10.3389/fimmu.2024.1426064

**Published:** 2024-06-17

**Authors:** Jia Tang, Chenming Shang, Yue Chang, Wei Jiang, Jun Xu, Leidan Zhang, Lianfeng Lu, Ling Chen, Xiaosheng Liu, Qingjia Zeng, Wei Cao, Taisheng Li

**Affiliations:** ^1^ Department of Infectious Diseases, Peking Union Medical College Hospital, Chinese Academy of Medical Sciences & Peking Union Medical College, Beijing, China; ^2^ Tsinghua Shenzhen International Graduate School, Tsinghua University, Shenzhen, China; ^3^ Department of Medical ICU, Peking Union Medical College Hospital, Chinese Academy of Medical Sciences & Peking Union Medical College, Beijing, China; ^4^ Department of Emergency Medicine, Peking Union Medical College Hospital, Peking Union Medical College, Chinese Academy of Medical Sciences, Beijing, China; ^5^ School of Medicine, Tsinghua University, Beijing, China; ^6^ Institute of Medical Information, Chinese Academy of Medical Sciences & Peking Union Medical College, Beijing, China; ^7^ State Key Laboratory of Complex Severe and Rare Diseases, Peking Union Medical College Hospital, Chinese Academy of Medical Science and Peking Union Medical College, Beijing, China; ^8^ Department of Basic Medical Sciences, School of Medicine, Tsinghua University, Beijing, China; ^9^ Tsinghua-Peking Center for Life Sciences, Beijing, China

**Keywords:** sepsis, mortality, immunophenotype, NK cells, machine-learning

## Abstract

**Background:**

Unbalanced inflammatory response is a critical feature of sepsis, a life-threatening condition with significant global health burdens. Immune dysfunction, particularly that involving different immune cells in peripheral blood, plays a crucial pathophysiological role and shows early warning signs in sepsis. The objective is to explore the relationship between sepsis and immune subpopulations in peripheral blood, and to identify patients with a higher risk of 28-day mortality based on immunological subtypes with machine-learning (ML) model.

**Methods:**

Patients were enrolled according to the sepsis-3 criteria in this retrospective observational study, along with age- and sex-matched healthy controls (HCs). Data on clinical characteristics, laboratory tests, and lymphocyte immunophenotyping were collected. XGBoost and k-means clustering as ML approaches, were employed to analyze the immune profiles and stratify septic patients based on their immunological subtypes. Cox regression survival analysis was used to identify potential biomarkers and to assess their association with 28-day mortality. The accuracy of biomarkers for mortality was determined by the area under the receiver operating characteristic (ROC) curve (AUC) analysis.

**Results:**

The study enrolled 100 septic patients and 89 HCs, revealing distinct lymphocyte profiles between the two groups. The XGBoost model discriminated sepsis from HCs with an area under the receiver operating characteristic curve of 1.0 and 0.99 in the training and testing set, respectively. Within the model, the top three highest important contributions were the percentage of CD38^+^CD8^+^T cells, PD-1^+^NK cells, HLA-DR^+^CD8^+^T cells. Two clusters of peripheral immunophenotyping of septic patients by k-means clustering were conducted. Cluster 1 featured higher proportions of PD1^+^ NK cells, while cluster 2 featured higher proportions of naïve CD4^+^T cells. Furthermore, the level of PD-1^+^NK cells was significantly higher in the non-survivors than the survivors (15.1% vs 8.6%, *P*<0.01). Moreover, the levels of PD1^+^ NK cells combined with SOFA score showed good performance in predicting the 28-day mortality in sepsis (AUC=0.91,95%CI 0.82–0.99), which is superior to PD1^+^ NK cells only(AUC=0.69, sensitivity 0.74, specificity 0.64, cut-off value of 11.25%). In the multivariate Cox regression, high expression of PD1^+^ NK cells proportion was related to 28-day mortality (aHR=1.34, 95%CI 1.19 to 1.50; *P*<0.001).

**Conclusion:**

The study provides novel insights into the association between PD1^+^NK cell profiles and prognosis of sepsis. Peripheral immunophenotyping could potentially stratify the septic patients and identify those with a high risk of 28-day mortality.

## Introduction

Sepsis is a life-threatening organ dysfunction characterized by an unbalanced host’s inflammatory response to infection (1). In 2017, a global study on sepsis burden estimated 48.9 million sepsis cases and 11 million sepsis-related deaths worldwide, which represents almost 20% of all deaths globally (2, 3). Once patients with sepsis require admission to critical care units, one-third of patients do not survive within 28 days and mortality varies by age, comorbid status, number, and type of organ dysfunction (4, 5).

Immune dysfunction is a pivotal pathophysiological feature of sepsis that involves different types of immune cells and complex molecular regulation thereof (4). During sepsis, inflammation and immunosuppression may occur sequentially or concurrently. An initial surge of pro-inflammatory cytokines and acute phase reactants, including interleukin(IL)-6, IL-8, ferritin, and C-reactive protein (CRP), followed by a compensatory anti-inflammatory response marked by elevated level of IL-10 in plasma, decreased expression of HLA-DR, and heightened expression of exhaustion markers including PD-1, TIM-3, and neutrophils CD88, along with an elevated proportion of regulatory T cells (Tregs), ultimately leads to drained and dysfunctional lymphocytes (4, 6–9). This phenomenon is called ‘sepsis-induced immunosuppression’, which has been related to adverse outcomes and increased mortality (10, 11). In light of this, researchers are actively engaged in the exploration and evaluation of biomarkers or digital signatures associated with sepsis and its phenotypes, to enhance diagnostic efficiency and identify potential physiological pathways and therapeutic targets (12).

Traditional biomarkers could be considered to provide information in systemic inflammation, including host-response biomarkers e.g., CRP, and procalcitonin (PCT), and organ dysfunction evaluation, e.g. sequential organ failure assessment (SOFA). However, given the complexity of sepsis, these indicators are neither specific nor sensitive to sepsis (13), leaving still needs for more new biomarkers. Therefore, as the immune dysregulation triggered by sepsis has gained more attention, monitoring the immune status of septic patients could be potential and crucial for the assessment of the prognosis and timely protection of organ function (14). Most patients with sepsis have reduced lymphocytes. Nevertheless, most viable lymphocytes are in an unresponsive state. Specifically, the quality and quantity of T cells, B cells and NK cells were altered in sepsis patients, resulting in impaired differentiation and activation of these immune cells, as well as high expression of negative costimulatory molecules. Besides, an increased frequency of immunosuppression markers [such as BTLA^+^CD4^+^T cells (15), TIM-3^+^CD4^+^T cells (16) and Th17/Treg ratio (17)] in septic patients is associated with higher rates of mortality or secondary infection. Consequently, there is a urgent need to identify the key features of immune cell alterations, and to further stratify septic patients with higher risk based on immunophenotyping biomarkers.

In the study, we investigated the role of T cells and natural killer (NK) cells in sepsis, especially the role of PD-1^+^NK cells in sepsis. To overcome the complexity and multiparameter of immune network dynamics in sepsis, machine learning (ML) encompassing a class of mathematical methods were applied. ML could process information from large datasets to generate core knowledge and insights (18), which is required in comprehensive depiction of immune profiles (19). It could further enhance the predictive and prognostic accuracy (18, 20, 21). In the present study, we proposed for the first time the ML-based immunophenotypes in sepsis and their relationship with other inflammatory biomarkers. Furthermore, we identified the patients with increased risk of mortality based on immunological subtypes.

## Materials and methods

### Study design and subjects

This retrospective study was conducted at Peking Union Medical College Hospital (PUMCH) between June 2023 and December 2023. Adult patients diagnosed with sepsis according to Sepsis-3 criteria were included, with at least one test of the peripheral immune cells (1). Exclusion criteria included patients aged less than 18 years, pregnant, or lack of immune cell testing. To better describe the predictive value of the immune patterns, septic patients with underlying conditions including chronic infection, autoimmune diseases, or cancers, were also included in our study set. Meanwhile, healthy controls (HCs) were included with age and sex matched with the septic patients.

### Data collection and flow cytometry

Clinical characteristics, including age, sex, sequential organ failure assessment (SOFA) score, underlying diseases, immunosuppressive drugs, infection sources and 28-day mortality, were retrospectively collected from medical records. Initial inflammatory tests upon admission were recorded, including complete blood count, hsCRP, PCT, IL-6, IL-8, and IL-10.

Immunophenotyping of peripheral blood lymphocytes was analyzed by 18-colour flow cytometry (LSRFortessa & trade; BD Biosciences, USA) as previously described (22). Fresh whole blood was tested with a panel of monoclonal antibodies against CD3/CD8/CD4, CD3/CD16 plus CD56, HLA-DR/CD38/PD-1/Ki-67/CD56 plus CD16, HLA-DR/CD38/PD-1/Ki-67/CD8, CD28/CD8/CD4, CD62L/CD45RA/CD4, CD25/CD127/CD4, and isotype controls (Immunotech, France). Cell surface marker expression was analyzed using Flowjo software v10.6. Cell counts of lymphocyte subsets were calculated with the white blood cell counts and lymphocyte differentials obtained from blood routine tests of the same specimen. Immunophenotyping methodologies and the threshold of expression of immune cells has been shown in **Supplementary Figure 1**.

### Machine learning

#### XGBoost

To discriminate immune cell profiles between septic patients and HCs, we utilized both the Logistic Regression and XGBoost (eXtreme Gradient Boosting) approaches, renowned for their established effectiveness in diverse machine learning prediction tasks. The logistic regression algorithm was implemented using the Scikit-Learn package (v1.0.2) in Python. Default values were utilized for the key hyperparameters in the LogisticRegression model. For the XGBoost approach, the XGBoost package (v1.6.2) in Python was employed. The key hyperparameters in the XGBoost model included the number of trees (n_estimators=1000) and maximum tree depth (max_depth=2), while the remaining hyperparameters adopted their default values.

To evaluate the performance of the models, the areas under the receiver operating characteristic curve (AUC) were computed. Furthermore, the SHapley Additive exPlanations (SHAP) values were calculated using the SHAP package (v0.42.1) in Python. These values were employed to visualize the interpretation of the contributing variables in both the Logistic Regression and XGBoost models.

#### K-means clustering

We used k-means clustering exploring the stratification of the immune cell profile in those sepsis patients. The number of optimum values for k (the number of clusters) was determined by the “NbClust” package in R (23), which turned out to be two clusters. After standardizing the proportions of T cell subsets and NK cells, K-means clustering was done.

#### PLAS-DA

To validate the features identified by XGBoost, we conducted subsequent partial least squares discriminant analysis (PLSDA) using the Scikit-Learn package in Python. This analysis employed ten-fold cross-validation with 5 repetitions to mitigate the risk of overfitting. We opted for PLS-DA due to its capability as a supervised machine learning tool, serving both feature selection and classification purposes. The prediction interval of the model was visually represented by the 95% confidence ellipses constructed between the two principal components.

### Statistical analysis

Statistical analyses were performed using R 4.1.3 (https://www.r-project.org/). Continuous data were reported as the mean± standard deviation (SD) or median (interquartile range [IQR]) depending on their normality, analyzed with Student’s t-test or rank-sum test as appropriate. Categorical data were reported as the number and percentage and compared using Fisher’s exact test or chi-square test. For correlation analysis, Pearson or Spearman analyses were performed as appropriate. The receiver operating characteristic (ROC) curve was used to determine the ability of lymphocyte subsets to discriminate septic patients from HCs, as well as comparing different biomarkers with 28-day mortality with Youden index. Immune cell levels were then classified using cutoff values. Cox regression analysis was conducted to determine the association of the potential biomarker with the 28-day mortality represented by the odds ratio (HR) and the 95% confidence intervals (CI). All tests were two-sided, and statistical significance was set at *P <*0.05.

## Results

### The lymphocyte profile in the peripheral blood in patients with sepsis was greatly disturbed

A total of 100 septic patients and 89 age and sex-matched HCs were enrolled. The median ages of the two groups were similar (59 [40.8–68] years in the sepsis group vs. 53 [39–61] years in the HC group), which means the immune profile could be comparable due to exception of age and sex interference.

The main characteristics of septic patients are shown in **Table 1**. In general, lungs were the main site of infection (n = 40; 40%), followed by intrabdominal infection (n = 28; 28%) and endocarditis (n = 20; 20%). Around 52% patients required mechanical ventilation, 55% required vasopressive drugs, and 22% developed AKI including 15% that required CRRT. Among sepsis patients, 22 patients (22%) died during the 28-day follow-up period.

**Table 1:** Baseline characteristics of the patients according to 28-day survival.

Parameters	All patients (n = 100)	Survivors (n = 78)	Non-Survivors (n = 22)	*P*
Demographic characteristics				
Age (years)	59.00 (40.75, 68.00)	58.00 (40.00, 67.00)	63.00 (58.00, 70.25)	0.197
Male, n (%)	64 (64.00)	50 (64.10)	14 (63.64)	0.968
Lab tests				
WBC (×10^9^/L)	10.54 (5.45, 14.66)	10.19 (5.98, 15.14)	11.41 (4.81, 13.15)	0.687
Lym%	9.25 (4.50, 19.62)	10.30 (4.62, 20.28)	7.25 (3.68, 13.35)	0.130
N%	79.90 (68.07, 88.93)	75.05 (64.67, 85.93)	88.70 (81.08, 90.88)	0.001
hsCRP (mg/L)	160.00 (96.80, 252.00)	157.00 (92.00, 243.00)	223.00 (133.77, 270.53)	0.133
PCT (ng/ml)	4.00 (0.80, 14.00)	4.60 (0.72, 15.50)	2.90 (1.65, 9.82)	0.896
IL-6 (pg/mL)	119.20 (36.64, 309.50)	127.48 (41.62, 322.50)	110.00 (36.00, 161.50)	0.520
IL-8 (pg/mL)	82.60 (44.50, 142.73)	78.00 (52.00, 131.46)	92.80 (26.72, 200.40)	0.990
IL-10 (pg/mL)	14.55 (7.35, 22.83)	14.80 (8.07, 22.78)	9.14 (5.00, 35.52)	0.534
Underlying diseases				
hypertension, n (%)	34 (34.00)	29 (37.18)	5 (22.73)	0.206
diabetes, n (%)	29 (29.00)	19 (24.36)	10 (45.45)	0.054
cancers, n (%)	11 (11.00)	8 (10.26)	3 (13.64)	0.951
Immunocompromised, n (%)	20 (20.00)	12 (15.38)	8 (36.36)	0.061
Steroids, n (%)	11 (11.00)	6 (7.69)	5 (22.73)	0.109
Immune Therapy, n (%)	15 (15.00)	9 (11.54)	6 (27.27)	0.137
Source of infection, n (%)				
Lung	40 (40.00)	23 (29.49)	17 (77.27)	<.001
Abdominal	28 (28.00)	24 (30.77)	4 (18.18)	0.246
Urinary Tract	9 (9.00)	6 (7.69)	3 (13.64)	0.661
Endocarditis	20 (20.00)	19 (24.36)	1 (4.55)	0.080
Others	10 (10.00)	8 (10.26)	2 (9.09)	1.000
Severity				
Shock, n (%)	54 (54.00)	35 (44.87)	19 (86.36)	<.001
SOFA, M (Q_1_, Q_3_)	4.00 (2.00, 9.00)	3.00 (2.00, 7.00)	11.00 (9.00, 12.00)	<.001
SOFA>8, n (%)	32 (32)	12 (15.38)	20 (90.91)	<.001
Vasopressive drugs, n (%)	55 (55.00)	36 (46.15)	19 (86.36)	<.001
Mechanical ventilation, n (%)	52 (52.00)	34 (43.59)	18 (81.82)	0.002
Acute kidney injury, n (%)	22 (22.00)	15 (19.23)	7 (31.82)	0.333
CRRT, n (%)	15 (15.00)	10 (12.82)	5 (22.73)	0.417
ICU length of stay, M (Q_1_, Q_3_)	2.00 (0.00, 10.00)	1.50 (0.00, 8.00)	5.00 (2.25, 12.25)	0.040
Length of hospitalization, M (Q_1_, Q_3_)	15.50 (8.75, 27.00)	17.00 (10.50, 28.75)	9.00 (3.00, 15.75)	0.001

Data are shown as median and interquartile range.

WBC, white blood cell; Lym%, the percentage of lymphocytes in WBC; N%, the percentage of neutrophils in WBC; SOFA sepsis-related organ failure assessment; hsCRP, high-sensitivity C-reactive protein; PCT, Procalcitonin; IL-6, interleukin-6; IL-8, interleukin-8; IL-10, interleukin-10; CRRT, continuous renal replacement therapy; ICU, Intensive Care Unit.

The lymphocyte profiles of septic patients exhibited notable distinctions compared with HC. The proportions of most cell subsets showed marked differences between sepsis and HCs. Proportions of Tregs, naïve CD4^+^T cells, PD1^+^CD4^+^T cells, Ki67^+^ CD4^+^T cells, CD38^+^DR^+^ CD4^+^T cells, PD1^+^ CD8^+^T cells, Ki67^+^ CD8^+^T cells, DR^+^ CD8^+^T cells, CD38^+^ CD8^+^T cells, CD38^+^DR^+^ CD8^+^T cells, DR^+^NK cells, CD38^+^NK cells, PD1^+^NK cells, and Ki67^+^NK cells in septic patients were significantly higher than those in HCs. In contrast, the absolute lymphocytes, memory CD4^+^T cells, and NK cells proportions were significantly lower in septic patients than those in HCs (**Supplementary Table 1**). These results indicated that patients with sepsis had a disrupted immune cell profile in the peripheral blood compared with HCs.

### Machine learning approach differentiates lymphocyte profiles between sepsis and HC

**Figure 1:**
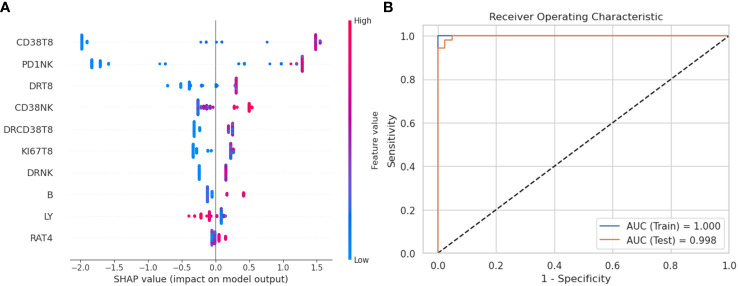
XGBoost model and SHAP value to evaluate variables’ importance. **(A)** SHAP summary plot to visualize the features’ impact on the model. The proportions of immune cell are ranked by importance (most important on top). The SHAP values on the x-axis indicate strength and direction of impact (positive value indicates increased probability of belonging to the sepsis group, a negative value indicates increased likelihood of belonging to the healthy control group). The color of the dots represents the feature value of corresponding immune cell proportions (blue if low, red if high). **(B)** Receiver operating characteristic (ROC) curve of XGBoost model in the training set and the testing set. SHAP, SHapley Additive explanation; XGBoost, eXtreme Gradient Boosting.

To further define and validate the immunophenotype in sepsis, we employed the XGBoost approach with the lymphocyte subsets from the peripheral blood of septic patients and HCs. The percentage of lymphocyte subsets containing 21 features entered the XGBoost model. After optimization, the accuracy of the XGBoost model in the testing set to distinguish septic patients from HCs was 1.0. Besides, the XGBoost algorithm showed AUC of 100% and 99.8% with ROC analysis in the training and testing set, respectively, indicating the outstanding effectiveness of this model in discriminating septic patients from HCs (**Figure 1B**).

To better visualize and explore the role of specific lymphocyte subsets in the XGBoost model, the top ten mean absolute SHAP scores were calculated and demonstrated in **Figure 1A**. The percentage of CD38^+^CD8^+^T cells, PD-1^+^NK cells, HLA-DR^+^ CD8^+^T cells represented the top three highest SHAP value contributions in the segregation of patients with sepsis from HCs in the XGBoost model.

For further validation, we carried the logistic model explaining the feature importance with SHAP and PLS-DA analysis (**Supplementary Figure 2**).

### The correlation between different lymphocyte subsets and serum biomarkers in sepsis

**Figure 2:**
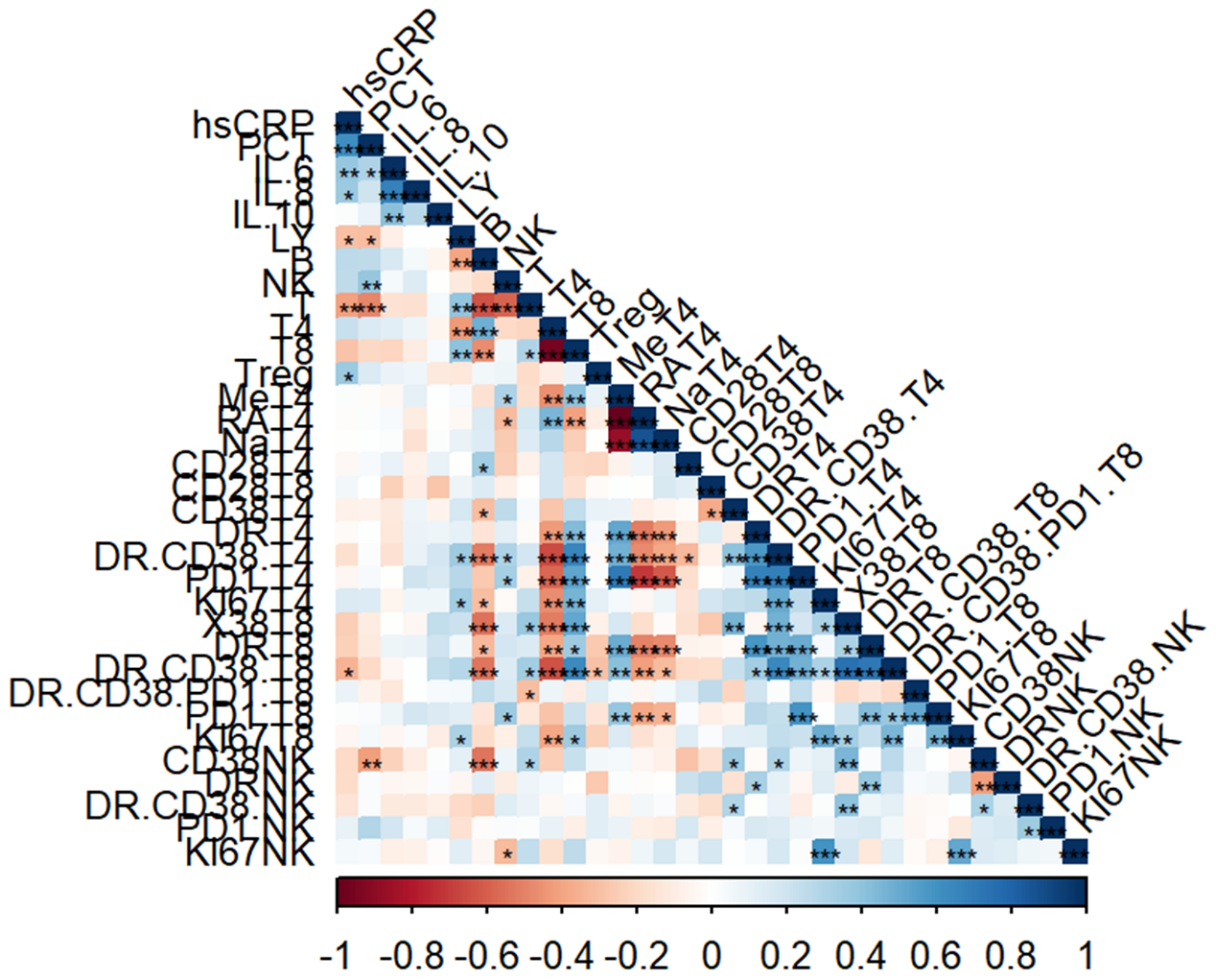
Correlation analysis of immune cell subsets and laboratory parameters. Heatmap representing the correlation analysis between percentage of immune cells with laboratory parameters (*, p<0.05, **, p<0.01, ***, p<0.001).

We then did correlation analysis to investigate the potential relationship between immune cell profile and serum biomarkers in sepsis. The heatmap showed the correlation of the proportions of different immune cell subsets with the serum biomarker in patients (**Figure 2**).

The percentage of lymphocytes were negatively correlated with hsCRP (r = -0.2911, *P* = 0.0497) and PCT (r= -0.3034, *P*= 0.0404). In addition, the percentage of CD3^+^ T cells also showed the same negative trend with hsCRP (r = -0.4066, *P* = 0.0050) and PCT (r= -0.4802, *P*=0.0007). As for NK cell subsets, the percentage of NK cells was positively correlated with PCT (r = 0.3895, *P* = 0.0075).

### The stratification of sepsis based on immune cell profile using the machine-learning approach revealed two clusters

**Figure 3:**
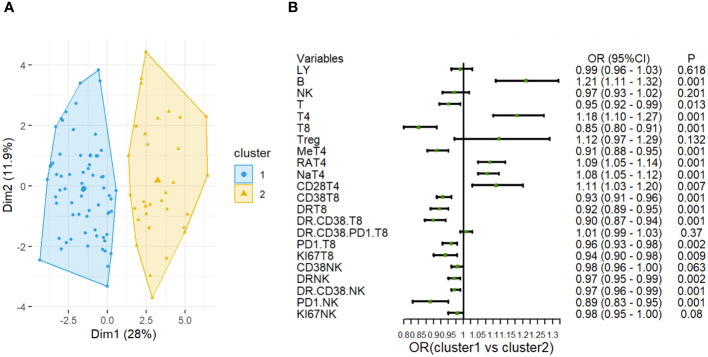
Cluster analysis based on the lymphocyte subsets in sepsis. **(A)** Clustering visualization (k = 2) obtained from k-means clustering. **(B)** Forest plot shows odds ratios with 95% CIs for the associations of lymphocyte cell subsets in Cluster 1 and Cluster 2.

To stratify septic patients based on immune cell profile, we utilized the ML approach of K-means clustering. Considering the immune cell recruitment in the sepsis patients, we focused on CD8^+^T and NK cell profiles. After confirming that two clusters were optimal, we did a k-means clustering analysis to investigate immune patterns among T cell subsets and NK cells (**Figure 3A**). As shown in **Supplementary Figure 3**, the cluster 1 (n = 32) was characterized by significantly higher proportions of PD1^+^NK cells (13.80 [8.32–21.75] % vs. 8.38 [5.02–12.80] %, *P*=0.001), CD38^+^CD8 T cells(79.25 [63.20 - 89.05] % vs.40.45 [31.38 - 60.75] %, *P*<0.001), HLA-DR^+^ CD8 T cells (76.60 [59.12 - 81.55] % vs.40.45 [(24.13 - 56.00] %, *P*<0.001), CD38^+^HLA-DR^+^CD8^+^ T cells(56.00 [40.05 - 70.20] % vs. 20.75 [9.70 - 27.85] %, *P*<0.001) and memory T cell(75.25 [58.90 - 83.92] % vs. 50.60 [38.62 - 63.92] %, *P*<0.001). While the cluster 2 (n = 68) featured significantly higher proportions of CD4^+^T (32.95 [22.70 - 45.75] % vs. 59.35 [50.63 - 70.17] %, *P*<0.001), naïve CD4^+^T (18.95 [9.65 - 37.32] % vs. 42.90 [31.93 - 56.92] %, *P*<0.001), CD4^+^CD28^+^T cells (91.65 [88.02 - 96.15] % vs. 95.25 [91.83 - 97.98] %, *P*= 0.017).

Notably, the difference in PD-1^+^ NK cells proportions was most obvious between the two clusters (**Figure 3B**).

### PD-1^+^NK immune cell profile based stratification predicted mortality rate in 28-day in sepsis

**Figure 4:**
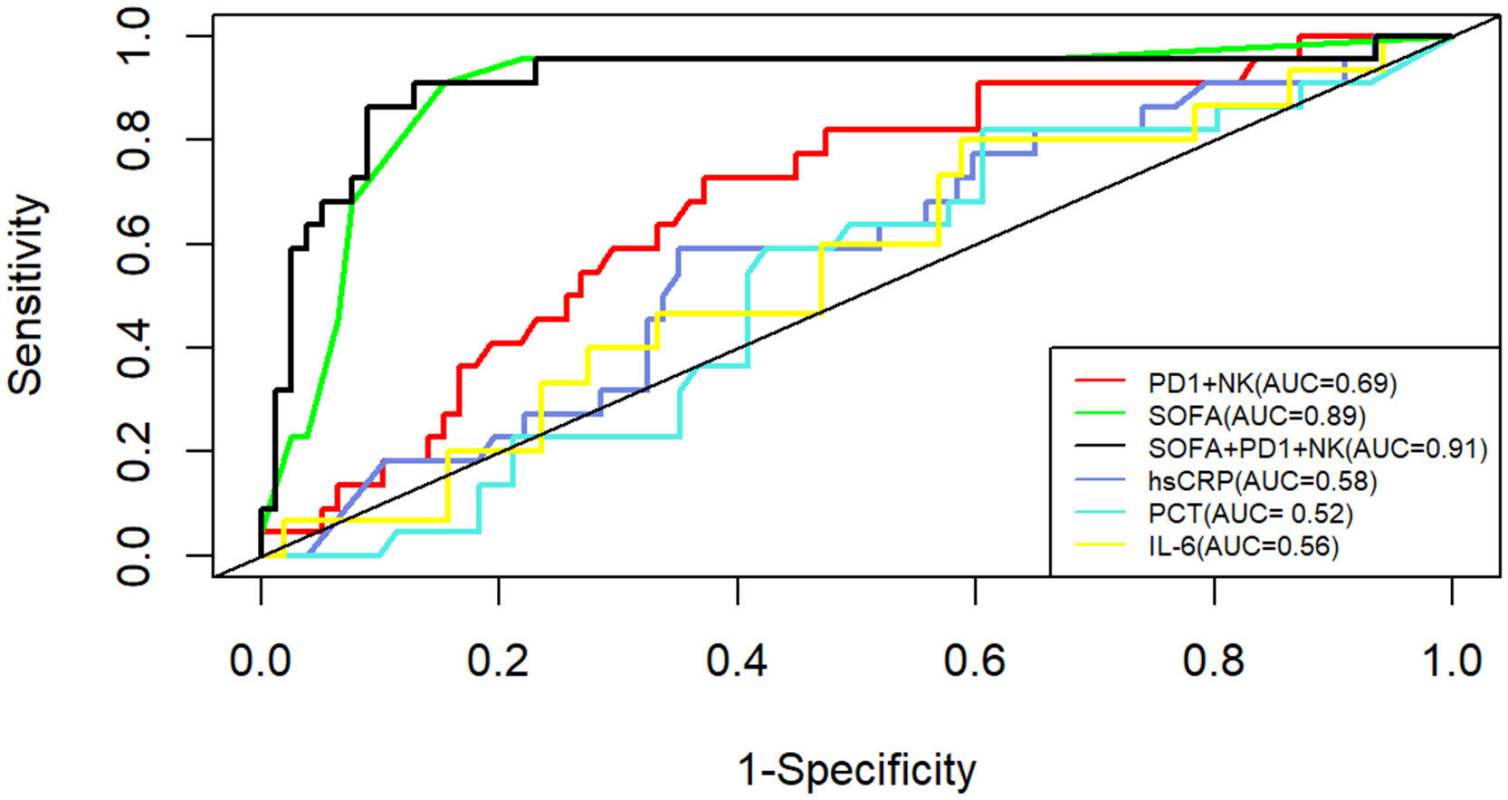
ROC curves of the PD-1+ NK levels, hsCRP, PCT, IL-6 levels and SOFA in predicting patients’ 28-day mortality. AUC, area under the curve; hsCRP, high-sensitivity C-reactive protein; SOFA, sepsis-related organ failure assessment.

Given the results of ML indicating a potential immune risk biomarker of PD-1^+^ NK cells, we further investigated the clinical utility of immunophenotypes in predicting the 28-day mortality for septic patients. As shown in **Supplementary Table 3**, the level of PD-1^+^NK cells were significantly higher in the non-survivors than those in the survivors (15.1% vs 8.6%, *P*<0.01). To further confirm the prognostic role of PD-1^+^NK cells in sepsis, we compared the predictive performance between PD-1^+^NK cells and commonly used inflammatory markers, including CRP, PCT, IL-6, as well as SOFA score. The area under the ROC curve (AUC) of the percentage of PD-1^+^NK cells and hsCRP, PCT and IL-6 for predicting 28-day mortality were 0.69 (0.58–0.80), 0.58 (0.45–0.71), 0.52(0.39–0.65), and 0.56 (0.39–0.72), respectively. Moreover, the SOFA score was 0.727 (0.635–0.807) (**Figure 4**). The AUC of the combination of percentage of PD-1^+^NK cells and SOFA score was 0.91 (0.82–0.99). The comparison of the AUC of SOFA+ PD-1^+^NK (the percentage of PD-1^+^NK cells) model vs. PD-1^+^NK (0.91 vs. 0.69, *P*<0.001), SOFA+PD-1^+^NK model vs. hsCRP (0.91 vs. 0.58, *P*<0.001), SOFA+PD-1^+^NK model vs. PCT(0.91 vs. 0.52, *P*<0.001), and SOFA+PD-1^+^NK model vs. IL-6 (0.91 vs. 0.56, *P*<0.001) indicates that the mortality prediction of the SOFA+ PD-1^+^NK model was better than the other isolated indicator. However, there was no statistical difference between SOFA+PD-1^+^NK model vs. SOFA score (0.91 vs. 0.89, *P*>0.05). The 28-day mortality was predicted according to the cutoff, and the sensitivity and specificity were shown in **Table 2**.

**Table 2:** The percentage of PD-1^+^ NK cells, SOFA score for predicting 28-day mortality.

Markers	Best cutoff	Specificity	Sensitivity	AUC	95%CI
hsCRP	194.5	0.64	0.56	0.579	0.451–0.707
PD1^+^NK	11.25	0.64	0.74	0.69	0.573–0.807
PCT	10.5	0.40	0.83	0.524	0.396–0.652
IL-6	194	0.41	0.8	0.556	0.392–0.719
SOFA	8.5	0.85	0.91	0.898	0.816–0.979

SOFA, sepsis-related organ failure assessment; PD-1 NK, the percentage of PD-1+ NK cell; hsCRP, high-sensitivity C-reactive protein; PCT, Procalcitonin; IL-6, interleukin-6.

Furthermore, based on the ROC analyses, patients were divided into high and normal groups of PD1^+^ NK cells proportion according to the cut-off value of 11.25%. We conducted univariate and multivariate Cox regression, and the regression result revealed a statistically predictive role of PD1^+^ NK cells in mortality of sepsis (HR=3.96, 95%CI 1.46 to 10.75; *P*=0.007). After adjusting for age, sex, and SOFA score, a multivariate Cox regression model also showed the increase in PD1^+^ NK cells proportion was related to 28-day mortality (aHR=1.34, 95%CI 1.19 to 1.50; *P*<0.001) (**Table 3**).

**Table 3:** Cox regression analysis for 28-day mortality.

Variables	Unadjusted Model		Adjusted Model	
	HR (95%CI)	*P*	aHR (95%CI)	*aP*
Age	1.02 (1.00 - 1.05)	0.108	3.78 (1.35 - 10.58)	0.011
SOFA	1.30 (1.18 - 1.43)	<.001	1.03 (1.00 - 1.06)	0.058
Male	1.51 (0.59 - 3.86)	0.389		
PD1^+^NK group				
<11.25	Ref		Ref	
≥11.25	3.96 (1.46 - 10.75)	0.007	1.34 (1.19 - 1.50)	<.001

SOFA, sepsis-related organ failure assessment; HR, hazard ratio; aHR, adjusted hazard ratio; Ref, reference.

## Discussion

Sepsis remains a devastating and life‐threatening clinical condition in practice. Due to its nature of dysregulated immune responses, more understanding of its immunological mechanism and potential biomarkers will better serve the clinical management and finally improve the prognosis. In this study, we conducted a comprehensive investigation of multiparametric immunophenotypes to determine immune dysfunction in septic patients and identify biomarkers for risks of mortality using machine learning approaches. We distinguished the prognostic values of PD-1^+^ NK cells in predicting 28-day mortality in sepsis. This was based on the following findings and arguments: (1) the percentage of PD-1^+^NK cells of sepsis patients who died within 28 days was significantly higher than that of those who survived, (2) multivariate cox regression analysis showed that the percentage of PD-1^+^ NK cells and SOFA score were independent risk factors for 28-day mortality, (3) the AUC of predicting the 28-day mortality was non-inferior to the ordinary inflammatory biomarkers. Our findings of the distinctive immune disturbances induced by sepsis suggested a possible role of immune modulating in improved outcomes of these critically ill patients (24).

In our study, we found that the immune phenotypes testing in peripheral blood could easily distinguish patients in sepsis with healthy controls, which indicated the necessity of immune evaluation in septic patients. The top three peripheral immune cell subtypes pinpointed by ML analysis in discriminating sepsis from HCs were CD38^+^CD8^+^T cells, PD-1^+^NK cells, and HLA-DR^+^ CD8^+^T cells. The circulating CD38^+^CD8^+^T cells and HLA-DR^+^ CD8^+^T cells represent the activation subsets of CD8^+^T cells. The persistent activation of NK and CD8^+^ T-lymphocytes plays a central role in eliminating pathogens in sepsis, similar as we depicted in COVID-19 in previous studies (22).

Not surprisingly, changes of NK cells have gained increased attention, acting as a significant risk factor for sepsis (25). They are the main effector cells in innate immunity which can recognize and attack viruses and bacteria (26). Previous studies have reported that the number of NK cells in the peripheral blood of sepsis patients is significantly higher than that of healthy individual (27). Programmed cell Death-1 (PD-1) is expressed on various immune cells (28). PD-1 overexpression in NK cell line resulted in decreased degranulation, indicating its suppressive effects not only on T cells but also on NK cells (29). Significant expression of PD-1 has been described in digestive cancers (30) and several infectious diseases, including chronic HIV (31), HBV (32), and HCV (33) infection, influenza (34), and SARS-CoV-2 infection (35), which contributes to an exhausted NK cell response.

However, less is considered on the prognostic value of PD-1 and other surface inhibitory receptors of NK cells in clinical studies, which might constrain the comprehensive understanding of NK cells in human sepsis. To the best of our knowledge, this is the first study investigating the prognostic role of the percentage of PD-1^+^ NK cells in sepsis. And we found that PD-1^+^ NK could predict the 28-day mortality of sepsis. Multiple clinical studies have established a correlation between PD-1 or PD-L1 expression and sepsis mortality (36). Previous studies mainly highlighted the high risk in mortality of patients with an increased expression of PD-1 by CD8^+^T cells or monocytes (8, 37, 38), or even combined exhausted CD8^+^ T cells pattern (2B4^hi^PD-1^hi^ CD160^low^ or 2B4^hi^ PD-1^low^ CD160^hi^) (39). However, comparisons of PD-1^+^NK cells and PD-1^-^ NK cells have revealed PD-1^+^NK cells to be functionally exhausted, with impaired cytotoxicity and cytokine production and reduced proliferative capability (28, 40). A prospective study pointed out that the expression of PD-1 in Tregs (OR:1.04;95%CI:1.00–1.07) and SOFA scores (OR:1.26;95%CI:1.05–1.52) were independent risk factors for 28-day mortality in septic patients (41). Furthermore, several studies proved that the percentage of monocytes and NK cells expressing PD-L1 can have discriminatory value for mortality with AUC values in the range of 0.66 to 0.85 (7, 42). Recently, an experimental study proved that high expression of PD-L1 were thought to be connected with sepsis progression, and the survival rate of septic mice was improved by anti-PD-L1 antibody treatment (43). These findings proved the significant role of the exhaustion pattern in terms of the critical illness in sepsis. Through evidence of over-stimulation by impaired cells deficient in MHC-I probably lead to upregulation of PD-1 expression on NK cells, we could propose that the impact of PD-1 blockade on NK cells, may be more nuanced in terms of impact on functional activity than CD8^+^T cell function, however, more evidence is still needed (25).

Furthermore, CRP and PCT levels did not predict 28-day mortality, which is similar to the earlier studies (42, 44). These results indicated that the percentage of PD-1^+^NK cells could predict the prognosis of sepsis patients earlier than conventional inflammatory markers, such as CRP and PCT. The AUC analysis showed that the percentage of PD-1^+^NK cells was similar to commonly used clinical SOFA scores in predicting the 28-day mortality. Observational studies have noted that levels of soluble programmed cell death ligand-1 (sPD-L1) in the peripheral blood are elevated in septic patients and positively correlated with CRP and PCT levels. However, these levels are not associated with poor prognosis at an early stage (45). Similarly, some immunosuppressive biomarkers like monocytic HLA-DR expression proved inadequate for predicting sepsis mortality in the initial phase. Additionally, a sustained decrease in mHLA-DR expression was noted in non-surviving patients (46).

In addition, two independent risk factors of regression analysis were constructed into a prediction model of SOFA+PD-1^+^NK model. Its performance in predicting 28-day mortality was significantly better than any other single indicator. The SOFA+PD-1^+^NK mode improves the AUC of SOFA scores (0.89–0.91) without the statistical difference, which may contribute to the excellent performance of SOFA in the study. The evaluation items of the SOFA score include the related indexes to evaluate the function of the nervous system, blood system, circulatory system, respiratory system, liver, and kidney, but exclude the immune function. The current results suggested that the inclusion of indicators reflecting the immune function into the SOFA scoring system might further optimize the predictive efficacy of SOFA scores.

As demonstrated above, circulatory immune cells play a critical role in the pathogenesis of sepsis. However, interpreting their role is complicated by the inherent limitations of observational studies and the complex interactions between host and bacteria, which render these studies susceptible to confounding factors and reverse causation. To address these challenges, statistical methods such as inverse probability weighting (IPW) and Mendelian randomization (MR) analysis are necessarily employed (47), albeit requiring large sample sizes. A recent study using MR analysis, based on genome-wide association studies (GWAS), identified causal effects of 36 and 34 immunophenotypes on sepsis and 28-day mortality, respectively (48). These findings supported the significant influence of immune cells in the pathogenesis of sepsis.

Our study has some limitations. First, the interpretation of our findings might be limited by the sample size and the fact that it was conducted at a single center. Further validation with a large clinical cohort is necessary. Second, ML algorithms act like a black box that can produce effective predictions, yet their complementary explanations are often invisible. Considering that, we attempted to utilize SHAP values to explain and visualize the weight of specific lymphocyte subsets in the ML model. Third, although routinely used at our institution, we acknowledge that achieving broader adoption across multiple centers remains a significant challenge. The modest contribution and feasibility of using this approach to enhance predictive performance in clinical settings could be further improved. Lastly, the functional studies related to inflammatory factors released by NK and T lymphocytes are not enrolled in the current study yet.

## Conclusion

In conclusion, our work showed that the expression of PD-1^+^NK cells was independent risk factors for 28-day mortality, and it may serve as valuable indicators for predicting prognosis of patients based on the sepsis-3.0 criteria. Whether to include an assessment of immune function in the SOFA score needs further investigation.

## Data availability statement

The original contributions presented in the study are included in the article/**Supplementary Material**. Further inquiries can be directed to the corresponding author.

## Ethics statement

The patients/participants provided their written informed consent to participate in this study. The study was approved by the Ethics Committee of Peking Union Medical College Hospital (Ethical Committee Number: I-23PJ1452). The patients/ participants provided their written informed consent to participate in this study.

## Author contributions

JT: Writing – original draft. CS: Methodology, Software, Writing – original draft. YC: Data curation, Resources, Writing – original draft. WJ: Data curation, Formal analysis, Resources, Writing – original draft. JX: Data curation, Formal analysis, Resources, Writing – original draft. LZ: Data curation, Methodology, Supervision, Writing – original draft. LL: Data curation, Methodology, Supervision, Writing – original draft. LC: Data curation, Formal analysis, Supervision, Writing – original draft. XL: Methodology, Supervision, Validation, Writing – original draft. QZ: Data curation, Formal analysis, Validation, Writing – original draft. WC: Conceptualization, Funding acquisition, Supervision, Writing – review & editing. TL: Conceptualization, Funding acquisition, Supervision, Writing – review & editing.
