# Supplementary figures and images for: Peripheral PD-1+NK cells could predict the 28-day mortality in sepsis patients
Source: Front Immunol. 2024 Jun 17;15:1426064. doi: 10.3389/fimmu.2024.1426064 (PMC11215063; doi:10.3389/fimmu.2024.1426064)

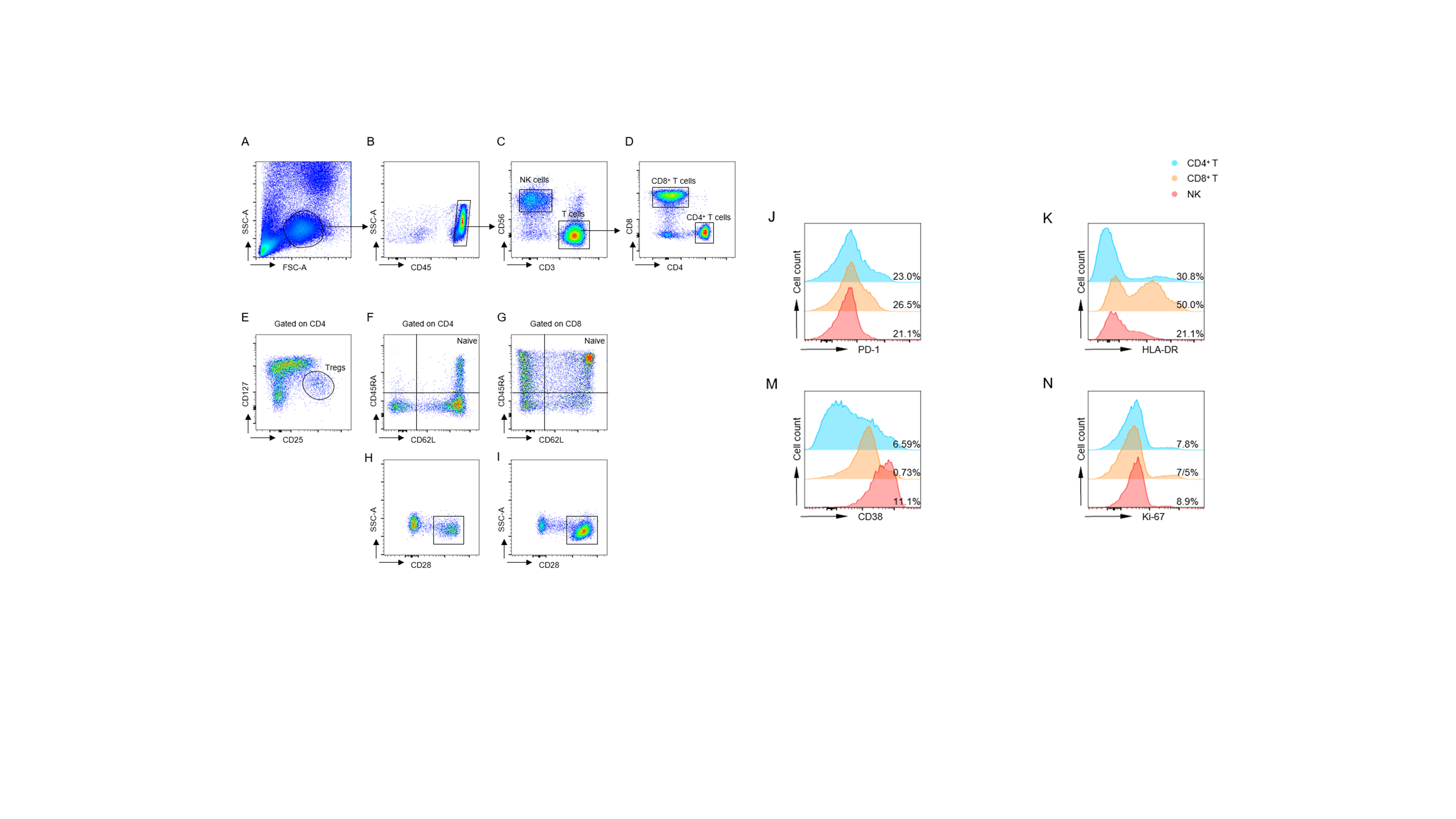

Supplement: Supplementary file 1 [file Image_1.tif]

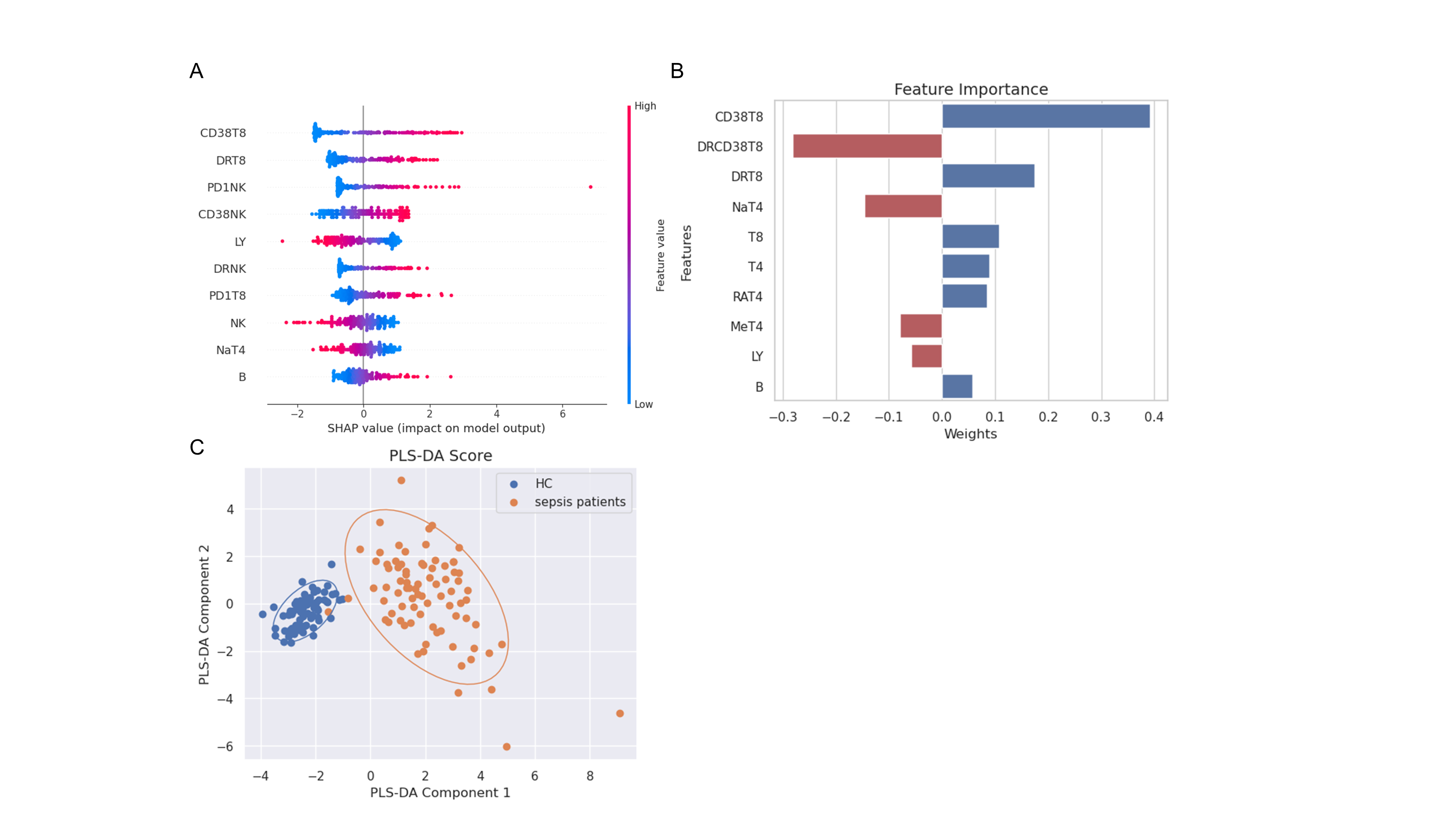

Supplement: Supplementary file 2 [file Image_2.tif]
