# Supplementary material for: Peripheral PD-1+NK cells could predict the 28-day mortality in sepsis patients
Source: Front Immunol. 2024 Jun 17;15:1426064. doi: 10.3389/fimmu.2024.1426064 (PMC11215063; doi:10.3389/fimmu.2024.1426064)
Supplement: Supplementary file 3 [file Table_1.docx]

Supplementary Material

**Peripheral PD-1+NK cells could predict the 28-day mortality in sepsis patients**

First Author*, Co-Author, Co-Author

Jia Tang*, Chenming Shang, Yue Chang, Wei Jiang, Jun Xu, Leidan Zhang, Lianfeng Lu, Ling Chen, Xiaosheng Liu, Qingjia Zeng, Wei Cao, Taisheng Li

*** Correspondence:** Wei Cao, Professor, Department of Infectious Diseases, Peking Union Medical College Hospital, Chinese Academy of Medical Sciences & Peking Union Medical College, Beijing 100730, China. (wcao_pumch@163.com); Taisheng Li, Professor, Department of Infectious Diseases, Peking Union Medical College Hospital, Chinese Academy of Medical Sciences & Peking Union Medical College, Beijing 100730, China. ([litsh@263.net](mailto:litsh@263.net))

Supplementary Figure 1. Gate strategy of immune cells

Flow cytometry analysis of expression of lymphocytes (A and B), NK and T cells (C), CD4+ and CD8+ T cells (D), Tregs (E), Naïve cells on CD4+ vs. CD8+ T cells(F-I), as well as the PD-1(J), HLA-DR(K), CD38(M) and Ki-67(N) expression on CD4+ T cells, CD8+ T cells and NK cells, respectively.

Supplementary Figure 2. Differences in immunologic architecture between sepsis and healthy control.

A: SHAP summary plot to visualize the features’ impact on the model through logistic model.

B: Factor-loading weights are shown for the top-ranked immune cell types with PLS-DA.

C: PLS-DA plot shows the difference of the immune cells between the HC and sepsis groups. Individual distribution points and confidence ellipses (ovals) in the plot represent for the HC and sepsis groups.

Supplementary Table 1. The comparison of lymphocyte subsets between sepsis and healthy controls in this study.

| **Variables** | **Total (n = 189)** | **Sepsis (n = 100)** | **HC (n = 89)** |  | ***P*** |
| --- | --- | --- | --- | --- | --- |
| Age, M (Q₁, Q₃) | 57.00 (40.00, 67.00) | 59.00 (40.75, 68.00) | 53.00 (39.00, 61.00) |  | 0.058 |
| Gender,male, n(%) | 109 (57.67) | 64 (64.00) | 45 (50.56) |  | 0.062 |
| LY, M (Q₁, Q₃) | 24.80 (7.60, 33.40) | 8.20 (4.35, 19.32) | 32.70 (28.40, 37.10) |  | <.001 |
| B, M (Q₁, Q₃) | 10.00 (6.63, 14.40) | 10.85 (4.90, 19.38) | 9.70 (7.50, 11.80) |  | 0.173 |
| NK, M (Q₁, Q₃) | 13.10 (8.00, 19.00) | 10.10 (5.40, 16.83) | 15.00 (11.00, 20.00) |  | <.001 |
| T, M (Q₁, Q₃) | 72.10 (61.90, 77.10) | 72.45 (62.05, 79.10) | 70.80 (61.90, 75.00) |  | 0.121 |
| T4, M (Q₁, Q₃) | 52.90 (44.90, 63.70) | 52.45 (41.67, 66.03) | 53.90 (45.80, 61.00) |  | 0.696 |
| T8, M (Q₁, Q₃) | 36.80 (29.00, 46.90) | 37.70 (27.00, 50.55) | 36.50 (30.40, 43.20) |  | 0.285 |
| Treg, M (Q₁, Q₃) | 7.00 (5.60, 8.80) | 8.14 (6.35, 10.00) | 6.20 (5.20, 7.30) |  | <.001 |
| MeT4, M (Q₁, Q₃) | 63.90 (51.50, 75.00) | 58.25 (41.88, 71.35) | 67.00 (57.00, 78.30) |  | <.001 |
| RAT4, M (Q₁, Q₃) | 36.30 (25.00, 48.50) | 41.75 (28.65, 58.28) | 33.60 (21.90, 42.90) |  | <.001 |
| NaT4, M (Q₁, Q₃) | 34.60 (20.80, 44.90) | 38.60 (21.20, 54.95) | 31.90 (20.80, 42.00) |  | 0.037 |
| CD28T8, M (Q₁, Q₃) | 60.10 (40.80, 76.20) | 66.70 (49.30, 85.25) | 53.10 (35.40, 65.00) |  | <.001 |
| CD38T8, M (Q₁, Q₃) | 18.20 (2.90, 53.20) | 51.05 (35.45, 74.00) | 2.70 (1.50, 5.20) |  | <.001 |
| DRT8, M (Q₁, Q₃) | 19.00 (8.70, 53.80) | 52.85 (31.57, 67.60) | 9.00 (5.50, 14.00) |  | <.001 |
| DRCD38T8, M (Q₁, Q₃) | 6.00 (1.60, 27.80) | 26.30 (15.45, 49.20) | 1.50 (0.80, 2.30) |  | <.001 |
| PD1T8, M (Q₁, Q₃) | 18.10 (12.00, 35.10) | 34.20 (22.03, 47.80) | 12.20 (9.00, 16.20) |  | <.001 |
| KI67T8, M (Q₁, Q₃) | 1.30 (0.60, 5.77) | 5.50 (2.85, 9.42) | 0.60 (0.40, 0.90) |  | <.001 |
| CD38NK, M (Q₁, Q₃) | 55.20 (34.40, 81.30) | 80.35 (58.75, 92.80) | 35.90 (23.80, 50.70) |  | <.001 |
| DRNK, M (Q₁, Q₃) | 11.10 (3.00, 43.30) | 39.45 (19.40, 58.10) | 3.00 (2.00, 5.40) |  | <.001 |
| PD1NK, M (Q₁, Q₃) | 2.94 (0.60, 11.20) | 10.45 (5.30, 15.53) | 0.50 (0.30, 1.00) |  | <.001 |
| KI67NK, M (Q₁, Q₃) | 1.80 (0.70, 5.37) | 4.89 (2.16, 10.75) | 0.90 (0.50, 1.60) |  | <.001 |

Note: HC, healthy control; M: Median, Q₁: 1st Quartile, Q₃: 3st Quartile

Supplementary Table 2. The comparison of lymphocyte cell subsets between Cluster 1 and Cluster 2 in sepsis.

| **Subsets** | **Total (n = 100)** | **cluster 1 (n = 32)** | **cluster 2 (n = 68)** | ***P*** |
| --- | --- | --- | --- | --- |
| LY, M (Q₁, Q₃) | 8.20 (4.35 - 19.32) | 11.60 (4.15 - 19.85) | 6.90 (4.47 - 17.85) | 0.440 |
| B, M (Q₁, Q₃) | 10.85 (4.90 - 19.38) | 4.17 (1.58 - 6.25) | 15.85 (9.33 - 21.95) | <.001 |
| NK, M (Q₁, Q₃) | 10.10 (5.40 - 16.83) | 13.15 (7.65 - 18.18) | 8.65 (5.38 - 15.40) | 0.065 |
| T, M (Q₁, Q₃) | 72.45 (62.05 - 79.10) | 77.85 (70.85 - 84.25) | 71.20 (60.55 - 75.53) | 0.005 |
| T4, M (Q₁, Q₃) | 52.45 (41.67 - 66.03) | 32.95 (22.70 - 45.75) | 59.35 (50.63 - 70.17) | <.001 |
| T8, M (Q₁, Q₃) | 37.70 (27.00 - 50.55) | 56.20 (48.17 - 67.90) | 31.10 (23.15 - 40.75) | <.001 |
| Treg, M (Q₁, Q₃) | 8.14 (6.35 - 10.00) | 7.65 (5.89 - 9.55) | 8.19 (6.67 - 10.22) | 0.247 |
| MeT4, M (Q₁, Q₃) | 58.25 (41.88 - 71.35) | 75.25 (58.90 - 83.92) | 50.60 (38.62 - 63.92) | <.001 |
| RAT4, M (Q₁, Q₃) | 41.75 (28.65 - 58.28) | 24.75 (16.07 - 40.75) | 49.40 (36.08 - 61.23) | <.001 |
| NaT4, M (Q₁, Q₃) | 38.60 (21.20 - 54.95) | 18.95 (9.65 - 37.32) | 42.90 (31.93 - 56.92) | <.001 |
| CD28T4, M (Q₁, Q₃) | 94.20 (90.75 - 97.53) | 91.65 (88.02 - 96.15) | 95.25 (91.83 - 97.98) | 0.017 |
| CD38T8, M (Q₁, Q₃) | 51.05 (35.45 - 74.00) | 79.25 (63.20 - 89.05) | 40.45 (31.38 - 60.75) | <.001 |
| DRT8, M (Q₁, Q₃) | 52.85 (31.57 - 67.60) | 76.60 (59.12 - 81.55) | 40.45 (24.13 - 56.00) | <.001 |
| DR CD38 T8, M (Q₁, Q₃) | 26.30 (15.45 - 49.20) | 56.00 (40.05 - 70.20) | 20.75 (9.70 - 27.85) | <.001 |
| DR CD38 PD1 T8, M (Q₁, Q₃) | 54.65 (41.95 - 72.17) | 50.10 (36.12 - 69.85) | 55.95 (44.80 - 72.17) | 0.375 |
| PD1 T8, M (Q₁, Q₃) | 34.20 (22.03 - 47.80) | 45.20 (28.75 - 58.15) | 30.85 (18.75 - 43.30) | 0.003 |
| KI67T8, M (Q₁, Q₃) | 5.50 (2.85 - 9.42) | 8.66 (5.33 - 16.45) | 4.40 (2.38 - 7.62) | <.001 |
| CD38NK, M (Q₁, Q₃) | 80.35 (58.75 - 92.80) | 91.40 (67.22 - 95.72) | 75.75 (54.65 - 87.88) | 0.009 |
| DRNK, M (Q₁, Q₃) | 39.45 (19.40 - 58.10) | 51.20 (38.60 - 71.93) | 28.35 (17.05 - 54.68) | 0.003 |
| DR CD38 NK, M (Q₁, Q₃) | 21.65 (13.70 - 41.70) | 35.95 (19.25 - 69.30) | 18.75 (10.12 - 36.95) | <.001 |
| PD1 NK, M (Q₁, Q₃) | 10.45 (5.30 - 15.53) | 13.80 (8.32 - 21.75) | 8.38 (5.02 - 12.80) | 0.001 |
| KI67NK, M (Q₁, Q₃) | 4.89 (2.16 - 10.75) | 8.45 (2.81 - 13.00) | 4.33 (1.78 - 7.44) | 0.066 |

Supplementary Table 3. The comparison of lymphocyte cell subsets between survivors and non-survivors in sepsis.

| **Subsets** | **Survivors (n = 78)** | **Non-Survivors (n = 22)** | ***P*** |
| --- | --- | --- | --- |
| LY, M (Q₁, Q₃) | 9.25 (4.70, 20.28) | 5.00 (2.65, 13.35) | **0.039** |
| B, M (Q₁, Q₃) | 10.90 (5.75, 19.15) | 10.65 (4.13, 20.40) | 0.690 |
| NK, M (Q₁, Q₃) | 10.75 (6.55, 16.70) | 5.84 (2.83, 16.90) | 0.055 |
| T, M (Q₁, Q₃) | 72.45 (61.60, 78.40) | 73.00 (63.42, 83.73) | 0.492 |
| T4, M (Q₁, Q₃) | 53.20 (45.08, 65.70) | 49.35 (28.82, 68.60) | 0.461 |
| T8, M (Q₁, Q₃) | 37.00 (27.32, 48.17) | 46.25 (27.52, 62.60) | 0.274 |
| Treg, M (Q₁, Q₃) | 8.30 (6.41, 10.15) | 7.75 (6.28, 8.75) | 0.519 |
| MeT4, M (Q₁, Q₃) | 58.30 (44.08, 71.03) | 49.30 (39.10, 72.62) | 0.351 |
| RAT4, M (Q₁, Q₃) | 41.20 (28.98, 55.92) | 50.70 (27.45, 60.30) | 0.362 |
| NaT4, M (Q₁, Q₃) | 37.50 (21.40, 49.67) | 41.50 (21.78, 56.55) | 0.606 |
| 28T4, M (Q₁, Q₃) | 94.65 (91.00, 97.68) | 93.70 (89.60, 96.38) | 0.441 |
| 38T8, M (Q₁, Q₃) | 52.85 (36.35, 73.45) | 45.85 (29.62, 77.85) | 0.569 |
| DRT8, M (Q₁, Q₃) | 50.40 (32.02, 65.88) | 53.25 (32.45, 68.73) | 0.711 |
| DR+CD38+T8, M (Q₁, Q₃) | 26.30 (17.02, 48.75) | 32.10 (10.25, 54.65) | 0.888 |
| DR+CD38+PD1+T8, M (Q₁, Q₃) | 51.50 (37.98, 71.00) | 63.25 (48.93, 77.88) | 0.120 |
| PD1 T8, M (Q₁, Q₃) | 32.85 (20.88, 46.80) | 36.25 (27.38, 49.45) | 0.650 |
| KI67T8, M (Q₁, Q₃) | 5.50 (2.95, 9.79) | 5.15 (2.60, 8.25) | 0.621 |
| 38NK, M (Q₁, Q₃) | 79.65 (59.08, 91.95) | 84.40 (59.82, 93.95) | 0.560 |
| DRNK, M (Q₁, Q₃) | 37.10 (16.08, 56.83) | 47.10 (29.97, 61.15) | 0.054 |
| DR+CD38+NK, M (Q₁, Q₃) | 21.30 (13.70, 40.90) | 23.50 (15.05, 46.80) | 0.373 |
| PD1 NK, M (Q₁, Q₃) | 8.60 (5.12, 14.60) | 14.05 (10.40, 17.73) | **0.009** |
| KI67NK, M (Q₁, Q₃) | 4.60 (2.00, 10.30) | 5.95 (2.85, 14.40) | 0.474 |

**Method details**

****1.Packages used in PLS-DA and Xgboost****

"import numpy as np\n",

"import pandas as pd\n",

"import shap\n",

"import xgboost as xgb\n",

"from sklearn.linear_model import LogisticRegression\n",

"from sklearn.model_selection import train_test_split\n",

"from sklearn.metrics import accuracy_score, roc_auc_score, roc_curve\n",

"import matplotlib.pyplot as plt\n",

"import seaborn as sns\n",

"from sklearn.cross_decomposition import PLSRegression\n",

"from sklearn.preprocessing import LabelBinarizer, StandardScaler\n",

"from matplotlib.patches import Ellipse\n",

"import matplotlib.transforms as transforms"

****2. Fit model****

]

},

{

"cell_type": "code",

"execution_count": null,

"metadata": {},

"outputs": [],

"source": [

"X_train, X_test, y_train, y_test = train_test_split(X, y, test_size=0.2)\n",

"scaler = StandardScaler()\n",

"X_train = scaler.fit_transform(X_train)\n",

"X_test = scaler.transform(X_test)\n",

"model = xgb.XGBClassifier(max_depth=2, n_estimators=1000, gamma=0.001, subsample=0.7, colsample_bytree=0.4, objective='binary:logistic', eval_metric='auc')\n",

"#model = LogisticRegression()\n",

"model.fit(X_train, y_train)"

]

},

{

"cell_type": "markdown",

"metadata": {},

"source": [

****3. Plot the AUC****

**]**

},

{

"cell_type": "code",

"execution_count": null,

"metadata": {},

"outputs": [],

"source": [

"y_pred = model.predict(X_test)\n",

"accuracy = accuracy_score(y_test, y_pred)\n",

"y_pred_prob = model.predict_proba(X_test)[:, 1]\n",

"fpr, tpr, thresholds = roc_curve(y_test, y_pred_prob)\n",

"auc = roc_auc_score(y_test, y_pred_prob)\n",

"\n",

"y_train_pred_prob = model.predict_proba(X_train)[:, 1]\n",

"fpr_train, tpr_train, thresholds_train = roc_curve(y_train, y_train_pred_prob)\n",

"auc_train = roc_auc_score(y_train, y_train_pred_prob)\n",

"\n",

"fig, ax = plt.subplots()\n",

"ax.plot(fpr_train, tpr_train, label='AUC (Train) = %0.3f' % auc_train)\n",

"ax.plot(fpr, tpr, label='AUC (Test) = %0.3f' % auc)\n",

"ax.plot([0, 1], [0, 1], 'k--')\n",

"\n",

"ax.set_xlim([-0.05, 1.0])\n",

"ax.set_ylim([0.0, 1.05])\n",

"ax.set_xlabel('1 - Specificity')\n",

"ax.set_ylabel('Sensitivity')\n",

"\n",

"ax.legend(loc=\"lower right\")\n",

"ax.set_title('Receiver Operating Characteristic')\n",

"fig.savefig('roc.pdf', format='pdf', dpi=600, bbox_inches='tight')"

]

},

{

"cell_type": "markdown",

"metadata": {},

"source": [

****4. Use SHAP to explain the features****

]

},

{

"cell_type": "code",

"execution_count": null,

"metadata": {},

"outputs": [],

"source": [

"X = scaler.fit_transform(X)\n",

"explainer = shap.explainers.Tree(model, X)\n",

"#explainer = shap.explainers.Linear(model, X)\n",

"shap_values = explainer(X)\n",

"\n",

"fig, ax = plt.subplots(dpi=600)\n",

"sns.set_style('white')\n",

"shap.summary_plot(shap_values, X, feature_names=marker_names, max_display=10, title=\"SHAP Summary Plot\")\n",

"fig.savefig('shap.pdf', format='pdf', bbox_inches='tight')"

]

},

{

"cell_type": "markdown",

"metadata": {},

"source": [

**5. PLS-DA score feature importance**

]

},

{

"cell_type": "code",

"execution_count": null,

"metadata": {},

"outputs": [],

"source": [

"raw_data = pd.read_csv('bisubsets-new-delete.csv')\n",

"raw_data.columns = raw_data.columns.str.replace(' ', '')\n",

"marker_names = raw_data.columns[4:]\n",

"X = raw_data.iloc[:, 4:]\n",

"y = raw_data['group'] - 1\n",

"\n",

"lb = LabelBinarizer()\n",

"y_binarized = lb.fit_transform(y).ravel()\n",

"X_train, X_test, y_train, y_test = train_test_split(X, y_binarized, test_size=0.2, random_state=42)\n",

"\n",

"n_components = min(X_train.shape[1], 10)\n",

"plsda = PLSRegression(n_components=n_components)\n",

"plsda.fit(X_train, y_train)\n",

"\n",

"\n",

"y_pred = plsda.predict(X_test)\n",

"y_pred_class = (y_pred > 0.5).astype(int)\n",

"\n",

"feature_weights = plsda.coef_.ravel()\n",

"sorted_indices = np.abs(feature_weights).argsort()[::-1]\n",

"sorted_weights = feature_weights[sorted_indices]\n",

"sorted_features = marker_names[sorted_indices]\n",

"\n",

"data = {'Features': sorted_features[:10], 'Weights': sorted_weights[:10]}\n",

"df = pd.DataFrame(data)\n",

"\n",

"sns.set(style=\"whitegrid\")\n",

"plt.figure()\n",

"sns.barplot(x='Weights', y='Features', data=df, palette=['r' if w < 0 else 'b' for w in sorted_weights[:10]])\n",

"plt.xlabel('Weights')\n",

"plt.ylabel('Features')\n",

"plt.title('Feature Importance', fontsize=14)\n",

"plt.tight_layout()\n",

"plt.show()"

]

},

{

"cell_type": "markdown",

"metadata": {},

"source": [

**6. PLS-DA score confidence ellipse**

]

},

{

"cell_type": "code",

"execution_count": null,

"metadata": {},

"outputs": [],

"source": [

"def confidence_ellipse(x, y, ax, n_std=2.0, facecolor='none', **kwargs):\n",

" if x.size != y.size:\n",

" raise ValueError(\"x and y must be the same size\")\n",

" cov = np.cov(x, y)\n",

" pearson = cov[0, 1] / np.sqrt(cov[0, 0] * cov[1, 1])\n",

" radius_x = np.sqrt(1 + pearson)\n",

" radius_y = np.sqrt(1 - pearson)\n",

" ellipse = Ellipse((0, 0), width=radius_x * 2., height=radius_y * 2., facecolor=facecolor, **kwargs)\n",

"\n",

" scale_x = np.sqrt(cov[0, 0]) * n_std\n",

" scale_y = np.sqrt(cov[1, 1]) * n_std\n",

" mean_x = np.mean(x)\n",

" mean_y = np.mean(y)\n",

"\n",

" transf = transforms.Affine2D() \\\n",

" .rotate_deg(45) \\\n",

" .scale(scale_x, scale_y) \\\n",

" .translate(mean_x, mean_y)\n",

"\n",

" ellipse.set_transform(transf + ax.transData)\n",

" return ax.add_patch(ellipse)\n",

"\n",

"scaler = StandardScaler()\n",

"X_scaled = scaler.fit_transform(X)\n",

"\n",

"X_train, X_test, y_train, y_test = train_test_split(X_scaled, y, test_size=0.2, random_state=42)\n",

"\n",

"plsda = PLSRegression(n_components=2)\n",

"plsda.fit(X_train, y_train)\n",

"\n",

"scores_train = plsda.transform(X_train)\n",

"scores_test = plsda.transform(X_test)\n",

"fig, ax = plt.subplots()\n",

"\n",

"labels = ['HC', 'sepsis patients']\n",

"i = 0\n",

"for label, color in zip(np.unique(y), sns.color_palette()):\n",

" mask = y_train == label\n",

" ax.scatter(scores_train[mask, 0], scores_train[mask, 1], color=color, label=labels[i])\n",

" i += 1\n",

"\n",

"for label, color in zip(np.unique(y), sns.color_palette()):\n",

" mask = y_train == label\n",

" confidence_ellipse(scores_train[mask, 0], scores_train[mask, 1], ax, n_std=2, edgecolor=color)\n",

"\n",

"ax.set_xlabel('PLS-DA Component 1')\n",

"ax.set_ylabel('PLS-DA Component 2')\n",

"ax.legend(loc='upper right')\n",

"ax.set_title('PLS-DA Score', fontsize=14)\n",

"plt.show()"

]

}

],

"metadata": {

"kernelspec": {

"display_name": "normal",

"language": "python",

"name": "python3"

},

"language_info": {

"codemirror_mode": {

"name": "ipython",

"version": 3

},

"file_extension": ".py",

"mimetype": "text/x-python",

"name": "python",

"nbconvert_exporter": "python",

"pygments_lexer": "ipython3",

"version": "3.7.13"

},

"vscode": {

"interpreter": {

"hash": "c46f867eecf9cebc75116310492e9b80acfa9b791dcd21533c9f8f3e36d45b35"

}

}

},

"nbformat": 4,

"nbformat_minor": 2

}

**7. Related computerized programs for k-means clustering in R**

#library packages needed

library(mice)

library(factoextra)

library(dplyr)

library(cluster)

library(NbClust)

library(ggplot2)

## normalization

bc.scaled<-scale(filled)

# check the optimal number of clustering

result <- fviz_nbclust(df, kmeans, method = "wss")

# k-means clustering

set.seed()

k2<-kmeans(bc.scaled,2,nstart=25)

p1<-fviz_cluster(k2, data = filled,geom = "point")
